# Antimicrobial Properties of Extracellular Matrix Scaffolds for Tissue Engineering

**DOI:** 10.1155/2019/9641456

**Published:** 2019-12-13

**Authors:** Germán R. Jiménez-Gastélum, Elsa M. Aguilar-Medina, Eduardo Soto-Sainz, Rosalío Ramos-Payán, Erika L. Silva-Benítez

**Affiliations:** ^1^Faculty of Biology, Autonomous University of Sinaloa, Culiacan, Sinaloa 80010, Mexico; ^2^Faculty of Biological and Chemical Sciences, Autonomous University of Sinaloa, Culiacan, Sinaloa 80010, Mexico; ^3^Faculty of Odontology, Autonomous University of Sinaloa, Culiacan, Sinaloa 80010, Mexico

## Abstract

The necessity to manufacture graft materials with superior biocompatibility capabilities and biodegradability characteristics for tissue regeneration has led to the production of extracellular matrix- (ECM-) based scaffolds. Among their advantages are better capacity to allow cell colonization, which enables its successful integration into the tissue surrounding the area to be repaired. In addition, it has been shown that some of these scaffolds have antimicrobial activity, preventing possible infections; therefore, it could be used as an alternative to control surgical infection and decrease the use of antimicrobial agents. The purpose of this review is to collect the existing information about antimicrobial activity of the ECM and their components.

## 1. Introduction

Every year, millions of patients suffer traumatisms, diseases, or infections that lead to the loss of tissues such as skin [1], bone [2], nerves [3], cartilage [4], liver [5], and blood vessels, among others [6]. An option to treat these lesions is the use of grafts that provide mechanical, biological, and chemical support for cells [4], even though a common problem with these treatments is the microbial colonization despite the use of antimicrobials; their failure is due to the ineffectiveness in controlling the infection [7–9].

The gold standard material for bone regeneration is the autograft [10] since its use avoids the problem of immunological rejection. Unfortunately, to perform this procedure, it is necessary to perform a second surgical intervention, increasing the recovery time for the patient and the risk of infections [11]. When the graft-based treatments are applied, there are many opportunistic bacteria that can grow at the surgery site due to the lack of asepsis, resulting in an unsuccessful treatment, surgical removal of the graft, and economic loss [12]. For this reason, a variety of materials have been developed for their use as biological substitutes, seeking to improve these drawbacks [13–15].

A therapeutic strategy could be the use of decellularized tissue scaffolds; these have demonstrated to provide macro- and microenvironmental signals at compositional and structural level [16]. Currently, these scaffolds are produced from extracellular matrix (ECM) of a wide variety of tissues, including the small intestine submucosa (SIS) [17], urinary bladder matrix (UBM) [18], central nervous system [19], esophagus [20], liver [21], skeletal muscle [22], lung [23], umbilical cord [24], adipose tissue [25], dermis [26], colon [27], cartilage [28], and bone [29], among others.

The ECM is a complex of proteins [30], principally collagenous [31], that are exported out of the cell to assemble itself forming a 3D structure [32, 33] of well-organized elastic fibers, associated with reticular and collagen fibers; within an amorphous component composed of proteoglycans, glycosaminoglycans, such as heparan sulfate, chondroitin sulfate, and hyaluronic acid (HA), and multiadhesive glycoproteins that give space and support for the cells [34], to interact with the rest of the components of the ECM [35]. Its function is to counteract the crushing forces, absorbing large amounts of water and orienting the collagen fibers [36]. Collagen fibers interact with the elastic fibers which are composed mainly of elastin and fibrillin to prevent the tearing of the tissues for the stretch [37].

Depending on the origin of the ECM, it can also contain multiadhesive proteins such as fibronectin, laminin, ostenectin, osteocalcin, and osteopontin, among others. These facilitate the formation of 3D structures and allow the incorporation of growth factors [38]. As can be seen, the ECM-based scaffolds are made up of the same components as the extracellular environment, so they have the capacity to trigger the signaling pathways that promote survival, migration, proliferation, and cell differentiation [39, 40].

Additionally, there are evidences that proteins and peptides from the ECM, as well as other components attached to the ECM, can trigger antibacterial activity in vitro and in vivo. Due to its great properties to repair and exert antimicrobial activity, ECM can be considered an ideal material for tissue regeneration to prevent infections. This review will be focused on describing those ECM-based scaffolds, components, and bioactive peptides with antimicrobial activity (Table 1).

## 2. ECM-Based Scaffolds: Resistance to Bacterial Infection in Clinical Cases

ECM-based scaffolds from different sources have been tested for their capacity to control surgical infection, since it has been observed that this kind of graft has the capacity to generate antimicrobial peptides that protect the remodeling site [41, 45, 52], through a controlled release mechanism of enzymatic digestion [46, 53, 54]. This could lead to a new alternative to decrease the treatment with antimicrobials and improve the clinical prognosis.

### 2.1. Urinary Bladder Submucosa

Some researchers have evaluated the antimicrobial capacity of ECM-based scaffolds. Specifically, ECM from urinary bladder submucosa (UBS), decellularized with 0.1% paracetic acid and 4% ethanol, has demonstrated to be effective in controlling the bacterial growth in the repair of rat abdominal wall defects in the presence of *Staphylococcus aureus*. Analysis of blood cell counts and temperature of the evaluated animals showed normal levels at the second week postoperation, while histological analysis showed wide presence of mononuclear cells within a moderately organized collagenous connective tissue [44].

### 2.2. Small Intestinal Submucosa

A commercial graft of SIS (Surgisis®) has shown the ability to resist intentional bacterial contamination in the repair of laparotomy defects in rat. These scaffolds did not show any evidence of bacterial colonization of *S. aureus* or *Staphylococcus epidermidis*, and only few inflammatory cells with the evidence of host tissue remodeling were observed [42].

The antimicrobial capacity of Surgisis® was also evaluated in a colostomy porcine model with fecal stool contamination. Animals treated with Surgisis showed normal pulses and no signs of pseudoaneurysms. Despite the fact that one animal developed *Acinetobacter* infection, all grafts were incorporated to host tissue and endothelialized. Infiltration and proliferation of lymphocytes and fibroblasts were observed with no presence of neutrophils. Besides, production of collagen and elastin was detected inside the graft [55].

Furthermore, in a dog model of orthopedic soft tissue repair, another commercial SIS-based scaffold (RESTORE™) was evaluated in stifle joint defect deliberately infected with *S. aureus*. None of the dogs received antibiotics, and all animals increased their body weight. The macroscopic appearance showed healthy tissue, and the scaffold was well integrated into the host tissue since the adjacent tissue could not be identified. Microscopic appearance showed dispersed mononuclear cells into a well-organized and vascularized connective tissue. None of the positive cultures of the microorganism were obtained from the joint fluid of the dogs in the RESTORE™ group [43].

In a dog model, SIS-based scaffolds were obtained by a mechanical abrasion and sterilized in a 10% neomycin-saline solution for 15 minutes. These scaffolds did not show to trigger signs of fever after the first week, and the counts of leucocytes showed moderate presence of lymphocytes, macrophages, and neutrophils after an intentional *S. aureus* contamination. SIS-based scaffolds presented negative culture results, and the macroscopic examination showed mature granulation tissue with connective tissue incorporation around the scaffold without turbid fluid [52].

The mechanisms by which biological scaffolds materials composed of ECM resist to infections are not fully understood. However, there are evidences that ECM scaffold degradation is necessary [53, 54, 56].

## 3. Antimicrobial Properties within ECM from Different Tissues and Organs

Antimicrobial activity has been observed of ECMs derived from SIS, UBS, liver, dental pulp, and dentin. The components of these ECMs were obtained by different methods including boiling, enzymatic digestion, salt solubilization and precipitation, and chromatography. Intact ECM forms and ECM fragments have been tested not only against opportunistic bacteria but also against specific tissue-associated bacteria.

### 3.1. Small Intestine Submucosa

Normally, the small intestine is exposed to the presence of various bacteria and it is constantly producing antibacterial peptides to keep the growth of the microbiota controlled. It has been reported that fractions corresponding to 5–16 kDa in ECM extracts from SIS obtained by boiling with acetic acid solution and further size exclusion chromatography showed to inhibit the growth of *Escherichia coli* at concentrations of 0.77 mg/ml for up to 24 hours at microtiter plate, MIC assays. Also, this inhibitory effect was seen for *S. aureus* although this microorganism was less sensitive, demonstrating that the antibacterial activity varies among bacteria. However, there was a great difference between ECM extract groups compared with the negative control [41].

Nevertheless, other studies showed that SIS-based scaffolds did not exhibit antimicrobial properties. In disc diffusion susceptibility tests, a commercial graft is used (Surgisis®) against *Pseudomonas aeruginosa*, *Streptococcus pyogenes*, *E. coli*, *S. epidermidis*, and *S. aureus* (sensitive and resistant to methicillin). Grafts did not inhibit the growth of any bacteria. Interestingly, serial dilution assay with SIS-disc extracts from 1 cm^2^ segments in 0.85% of saline solution could inhibit the growth of *S. pyogenes* at 1 : 2, 1 : 8, and 1 : 16 dilutions without turbidity up to 24 hours [57]. Probably, these results are correlated with SIS-based scaffolds exposure to native 3D structure and not as extracts, though there are evidences that bacterial membrane composition might interfere with electrostatic interactions between peptides and bacterial surfaces. For example, *P. aeruginosa* PAO1 bacterial strain is reported to be susceptible due to the presence of 2-amino-2,6-dideoxy-D-galactopyranose on LPS and *P. aeruginosa* ATCC 27853 strain is resistant to the antimicrobial peptide (AMP) by the absence of LPS B-band [58]. Also, the presence of cardiolipin appears to be a determinant for AMP antibacterial activity against Gram-negative bacteria [59]. Likewise, DMS-DA6 peptides act by strong perturbation of the bacterial membrane against the Gram-positive bacteria *S. aureus* ATCC 6538 but not against the Gram-negative bacteria *E. coli* ATCC 35218 because of specificity DMS-DA6 interaction with peptidoglycan, a major component of the membrane of Gram-positive bacteria [60]. In this way, the resistance of *P. aeruginosa*, *E. coli*, *S. epidermidis*, and *S. aureus* to ECM extracts may result due to the kind of bacterial membrane composition.

### 3.2. Oral Tissues

Another region with a complex microbiota is the oral cavity. The bacteria that compose this microbiota can colonize several tissues, such as tongue, buccal and gingival epithelium, and dental organs [61].

To assess the antimicrobial capacity of dental pulp and dentin ECM, peptides from these ECM were purified by precipitation with ammonium sulfate (30, 40, 50, 70, and 90%). All of these fractions maintain its antibacterial activity against *Streptococcus mutans*, *Streptococcus oralis*, and *Enterococcus faecalis* at concentrations of 1, 5, and 10 *μ*g/ml; although after the initial 24 h of growth, the bacteria were cultured in fresh medium without ECM extracts showing only a bacteriostatic effect for both ECM extracts [47].

### 3.3. Urinary Bladder and Liver

The antibacterial activity of the ECM has not only been observed in tissues with microbiota but also in tissues that are not commonly colonized by bacteria such as the bladder and liver. Fractions of proteins from the ECM of these tissues were obtained through digestion and precipitation with ammonium sulfate. Protein concentrations of 40 and 90 mg/ml showed to inhibit *S. aureus* and *E. coli* growth [45].

The antibacterial activity of protein extracts of UBS was shown by the inhibition of the growth of *E. coli* and *S. aureus* at 1.60 mg/ml. The protein extracts strongly inhibit the growth of *E. coli*, while the growth inhibition of *S. aureus* showed a lower sensitivity, demonstrating that the antibacterial activity varies among bacteria [41].

### 3.4. Lung Extracellular Matrix

The antibacterial capacity of a scaffold decellularized with 0.1% SDS in PBS obtained from goat-lung was tested against Gram-negative (*E. coli*) and Gram-positive (*S. aureus*) bacteria. These bacteria were cultured in Mueller–Hilton (MH) broth until they reached an optical density of 0.1 at 570 nm. At this point, a collagenase—degraded of goat lung—matrix was added, showing antibacterial activity against *E. coli* for up to 9 h and *S. aureus* for up to 5 h. These results could be related to the releases of bioactive peptide molecules after enzymatic digestion and could help to provide immediate protection at the implantation site, until an immune response is activated [46].

## 4. Antimicrobial Peptides from Extracellular Matrix Compounds

The ECM is not only a tridimensional support for cells, it has also been demonstrated to have the capacity to regulate different cell activities [62, 63] through molecules named cryptic peptides. These are bioactive peptides originated by partial proteolysis of ECM macromolecules such as collagen, elastin, and some glycoproteins [62, 64]. These ECM macromolecules also contain bioactive regions with different functions and behavior than the parenting proteins [65]. Proteins contain short functional sequences inside their hydrophobic cores named cryptic peptides [64]. These have been related with antioxidant, cell adhesion, and angiogenic and arteriogenic functions [64] and are released after structural or conformational alterations derived by enzymatic degradation, multimerization, denaturation, adsorption, and cell-mediated mechanical forces [63].

### 4.1. Peptides Derived from Collagenous Protein

Collagen is one of the main proteins that constitute ECM, since it is involved in the formation of several fibers of the connective tissue. The alpha 3 subunit of collagen type VI has been showed to cause damage to the extracellular membrane and release of the cytoplasmic content of *S. aureus*, *E. coli*, and *P. aeruginosa* [48]. Also, it has been identified that the globular region of collagen type VI microfibrils (extracted from bovine cornea) interacts with the membrane of *Streptococcus* from A, B, and G groups lysing them at doses of 2 *μ*M, 100 nM, and 10 nM, respectively. In this process, it is essential the participation of surface adhesion M1-protein from the microorganism [49].

### 4.2. Peptides Derived from Noncollagenous Protein and Hyaluronic Acid

Peptides derived from protein such as fibronectin, laminin, and vitronectin display antimicrobial activity against Gram-positive and Gram-negative bacteria. The concentrations necessary to kill *E. faecalis*, *E. coli*, and *P. aeruginosa* varied between 0.3 and 3 *μ*M [50]. In the particular case of laminin, it has been demonstrated that peptides derived from *α*3 and *α*4 chains show a dose-dependent antibacterial activity against *S. aureus* and *E. coli*. This activity is related to the C-terminal globular region of the protein that is capable of permeating the extracellular membrane and binding to the bacterial DNA [66].

Microtiter plate-MIC assays which determine bactericidal or bacteriostatic effects of water-soluble HA evaluated on the growth of oral and no oral bacteria using HA of low (141 kD), medium (757 kD), and high (1,300 kD) molecular weight of 0.5, 1, and 2 mg/ml. These noncollagenous components showed a bacteriostatic activity against *S. mutans*, *Porphyromonas gingivalis*, *Prevotella oris*, *Aggregatibacter actinomycetemcomitans*, *S. aureus*, *and Propionibacterium acnes*, *A. actinomycetemcomitans* being the most inhibited. However, strains such as *S. mutans* and *P. gingivalis* showed inhibition or stimulation of growth depending on certain molecular weight and concentrations [51].

### 4.3. Peptides Derived from Growth Factors

A characteristic of ECM-scaffolds is that, after decellularization process, they maintain growth factors [44, 46]. It has been demonstrated that this kind of protein improves regeneration and also that growth factor-derived peptides such as platelet-derived growth factor (PDGF-A y PDGF-B), hepatocyte growth factor (HGC), heparin-binding EGF-like growth factor (HB-EGF), fibroblast growth factors (FGF), and amphiregulin exert bactericide activity against Gram-positive and Gram-negative bacteria [67].

### 4.4. Influence of Electrostatic Forces

It is notable that the antimicrobial activity is carried out by degradation products of the ECM components. The majority of these peptides have hydrophobic and basic amino acid sequences. Some peptides rich in hydrophobic amino acids from PRELP (proline-arginine-rich end leucine-rich repeat protein) and thrombospondin such as QPTRRPRPGTGPGRRPRPRPRP and KRFKQDGGWSHWSPWSS exert antimicrobial activity against Gram-positive and Gram-negative bacteria, respectively [57]. Also, the bioactive peptides derived from PDGF-A, PDGF-B, HGF, HB-EGF, FGF, and amphiregulin (GRPRESGKKRKRKRKLKPT, RVRRPPKGKHRKFKHTHDKTA, LKIKTKKVNTADQCANRCTRNKGL, GKRKKKGKGLGKKRDPCLRKYK, LKKNGSCKRGPRTHYGQKAIL, and PKRKKKGGKNGKNRRNRKKKN, respectively) are partially hydrophobic sequences and antimicrobial against *E.coli*, *P. aeruginosa*, and *Bacillus subtilis*, demonstrating a previously unknown activity of growth factor-derived peptides [67].

Cationic peptides present sequences such as SRNLSEIKLLISQARK, SRNLSEIKLLISQARKQAASIKVAVSADR, KDFLSIELFRGRVKV, KDFLSIELFRGRVKV derived from *α*1-chain, PPPPLTSASKAIQVFLLGGSRKRVL, LGTRLRAQSRQRSRPGRWHKVSVRW, RLRAQSRQRSRPGRWHKVSVRW, PGRWHKVSVRW from *α*5-chain, RIQNLLKITNLRIKFVKL from *β*1-chain of laminin, QPPRARITGYIIKYEKPG from fibronectin, AKKQRFRHRNRKGYR from vitronectin [41], and FAHIRDFVSRIVRR and FLLNTYRTKQEV that reside in the globular region of collagen type VI carried out an antimicrobial effect [48]. The bacterial membrane has a high negative charge due to its surface components, and therefore cationic peptides can interact with it [68].

It is reported that many of these AMPs kill bacteria by permeating their membranes. These AMPs contain a high load of hydrophobic and cationic amino acids; this allowed them to adopt an amphipathic *α*-helical, *β*-sheet, extended coil, or cyclic structure [69, 70]. Many *α*-helical AMPs can interact with components of the cellular wall such as lipopolysaccharides of Gram-negative bacteria or teichoic acid and peptidoglycans of Gram-positive bacteria and in both bacteria groups at the plasma membrane on phospholipid groups. These interactions can promote conformational changes, such as formation of an amphipathic helix and membrane destabilization, leading to a bacterial inactivation [71]. For example, in phosphate buffer, DMS-DA6-NH2 and DMS-DA6-OH show a random coil conformation of the peptides. In contrast, in the presence of negatively charged vesicles that mimic bacterial phospholipids, both peptides mostly adopted an *α*-helix conformation, indicating that electrostatic interactions between the cationic residues of DMS-DA6 and the negatively charged lipids play a major role in stabilizing the helical structure [60]; these conformational changes allow a peptide insertion at bacterial membrane to form pores [68]. Thus, secondary structure and electrostatic forces of the peptide undertake an antimicrobial activity.

**Figure 1:**
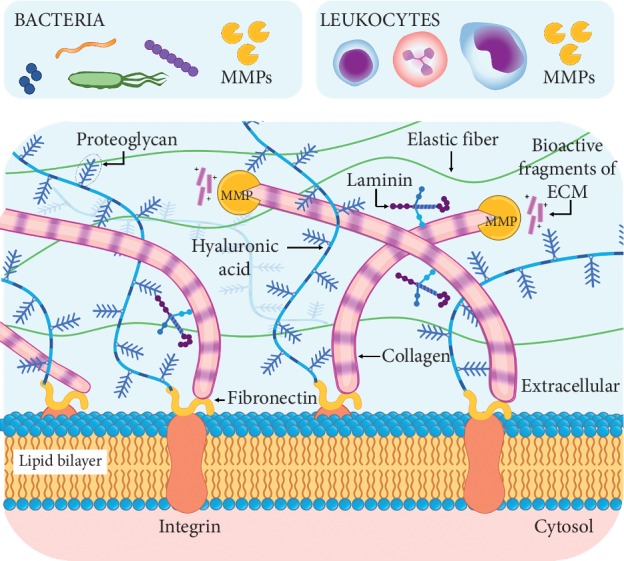
ECM degradation products. The interaction of bacteria and immune cells leads to ECM degradation through the release of matrix metalloproteinases generating bioactive fragments with antimicrobial effect.

The degradation of the scaffold is a primordial step prior to regeneration. In this process, degradation products of ECM develop an *α*-helical conformation and exert antimicrobial effect [50]. As seen in Figure 1, these bioactive fragments can be derived by the presence of matrix metalloproteinase (MMP) that can catalyze the cleavage of the ECM proteins [72, 73]. These MMPs are employed by several types of cells, including leucocytes such as neutrophils for migration through ECM [74, 75] and adipose-derived stem cells to increase angiogenesis [76]. Also, bacteria such as *P. gingivalis* can induce the release of MMP [77, 78]. This may explain why some ECM-based scaffolds can repair tissues in conditions of bacterial contamination in vivo [42–44, 52, 55].

## 5. Concluding Remarks

Many ECM components present antimicrobial activity against microorganisms that commonly contaminate surgical act and proliferate after this. Apparently, this effect resides on charged peptides that interact with the components of the cell surface and disrupt the cell activity. The antimicrobial activity depends on the microorganism species and type of components and concentrations of ECM that result in a bacteriostatic or bactericidal effect. There is evidence that these materials, employed for regeneration applications, can improve the outcome of in vivo experiment procedures. Nevertheless, it is necessary to continue the evaluation of these scaffolds against other pathogens, since most studies have focused on bacteria.

## Figures and Tables

**Table 1:** Antimicrobial properties within ECM.

ECM sources or ECM components	Bioactive peptides	Concentration	Sensitive strains	Tested assay	References
SIS	Scaffolds and extract	0.77 mg/ml	*E. coli*, *S. epidermidis*, and *S. aureus*	Graft infection and microtiter plate-MIC	[41, 42, 43]
UBS	Scaffolds and extract	1.60 mg/ml	*E. coli* and *S. aureus*	Graft infection and microtiter plate-MIC	[41, 44]
Liver	Extract	No reported	*E. coli* and *S. aureus*	Microtiter plate-MIC	[45]
Lung	Enzymatic degraded	25 *μ*L of 1 gr/ml	*E. coli* and *S. aureus*	Microtiter plate-MIC	[46]
Dental pulp	Extract	10 *μ*g/ml	*S. mutans*, *S. oralis*, and *E. faecalis*	Microtiter plate-MIC	[47]
Dentin	Extract	10 *μ*g/ml	*S. mutans*, *S. oralis*, and *E. faecalis*	Microtiter plate-MIC	[47]
Collagen	Extract	10 nM–2 *μ*M	*S. aureus*, *E. coli*, *P. aeruginosa*, and *Streptococcus* from A, B, and G groups	Microtiter plate-MIC and RDA	[48, 49]
Laminin	SRN16	0.6–100 *μ*M	*E. faecalis*, *E. coli*, and *P. aeruginosa*	Microtiter plate-MIC and RDA	[50]
Fibronectin	QPP18	10–100 *μ*M	*E. faecalis*, *E. coli* and *P. aeruginosa*	Microtiter plate-MIC and RDA	[50]
Vitronectin	AKK15	0.6–100 *μ*M	*E. faecalis*, *E. coli*, and *P. aeruginosa*	Microtiter plate-MIC and RDA	[50]
Hyaluronic acid	Hyaluronic acid (141, 757, and 1,300 kD)	2 mg/ml	*S. mutans*, *P. gingivalis*, *P. oris*, *A. actinomycetemcomitans*, *S. aureus*, *and P. acnes*	Microtiter plate-MIC and RDA	[51]
